# Awareness and Knowledge of the National Healthcare Transformation Program Among Public Health and Preventive Medicine Physicians in Saudi Arabia

**DOI:** 10.7759/cureus.66782

**Published:** 2024-08-13

**Authors:** Mubarak Aldawsari, Mohammed Alrowaily, Sara H Mustafa, Nawaf H Albali

**Affiliations:** 1 Preventive Medicine Residency Program, Riyadh Second Health Cluster, Riyadh, SAU; 2 Policy and Compliance, Council of Health Insurance, Riyadh, SAU; 3 Public Health, Epidemiology, and Biostatistics, College of Medicine, Alfaisal University, Riyadh, SAU

**Keywords:** saudi arabia, healthcare transformation, preventive medicine physicians, public health physicians, knowledge, awareness

## Abstract

Background: The Health Sector Transformation Program (HSTP) has been established as part of Saudi Vision 2030. Despite the significant progress achieved in previous years, the HSTP addresses multiple challenges. One of these challenges is that the current healthcare model prioritizes treatment over prevention. Therefore, prioritizing preventive healthcare measures is crucial.

Objectives:This study aims to assess the levels of awareness and knowledge among the public health and preventive medicine physicians in Saudi Arabia towards the national healthcare transformation program.

Method: In this cross-sectional study, we surveyed public health and preventive medicine physicians in Saudi Arabia. We collected data through a newly developed and validated self-administered questionnaire, which was distributed online. The survey questionnaire consisted of three sections: participants' background characteristics; measurement of public health and preventive medicine physicians' awareness of the HSTP and their confidence in their awareness; and measurement of the physicians' knowledge of the HSTP through six multiple-choice questions.

Results: In this study, 307 public health and preventive medicine physicians in Saudi Arabia participated. The mean age ± SD was 33.1 ± 5.1 years, with 54.7% being public health and preventive medicine residents, 33.2% specialists, and 12.1% consultants. Most participants showed high awareness of the HSTP and its strategic objectives. Over two-thirds correctly defined HSTP, value-based healthcare, and the new Model of Care (MOC), while about 50% demonstrated inadequate knowledge regarding Accountable Care Organizations (ACOs) and the "Keep Well" system. Older physicians, consultants, and those with more years of work experience had higher knowledge scores. Regression analysis revealed that job rank significantly influenced public health and preventive medicine physicians' knowledge of the HSTP.

Conclusion: This study shows that while public health and preventive medicine physicians are generally aware of HSTP's objectives, they have inadequate confidence in their knowledge, highlighting the need for better information dissemination. Awareness of the MOC is high, but gaps persist in understanding specific components like the "Keep Well" initiatives. The study also found that job rank and experience influence knowledge levels, suggesting the need for broader dissemination of the HSTP concepts.

## Introduction

Global health reform aims to improve health equity by addressing inequities in access to healthcare, strengthening health systems, and supporting universal health coverage. The core concepts of health reform include enhancing health infrastructure, investing in healthcare staff development, providing access to vital medications and technologies, and encouraging cross-sector collaboration. Primary healthcare, preventive interventions, and community-based initiatives are prioritized in addressing social determinants of health. Reforms also include policy improvements that improve healthcare financing, governance, and accountability. Effective health reform aims to build robust health systems capable of dealing with both routine healthcare requirements and public health emergencies [1].

Saudi Vision 2030 was unveiled in April 2016 with various strategic objectives and is based on three main pillars: a vibrant society, a thriving economy, and an ambitious nation [2]. The Health Sector Transformation Program (HSTP) has been established as part of Saudi Vision 2030 to realize the Kingdom's pillar of a "vibrant society" [3]. The HSTP will collaborate with all public and private health sector authorities to improve health and healthcare services, in line with Vision 2030's strategic national objectives [3]. The HSTP has four strategic objectives, which are increasing access to healthcare services, improving service quality and efficiency, promoting health risk prevention, and improving traffic safety [3, 4].

One of the HSTP's four strategic objectives is "promoting the prevention of health risks." This objective aimed at enhancing lifelong health, such as tackling social determinants of health and disparities, improving healthcare for an aging society, preventing chronic diseases, providing effective mental health care, and promoting overall population health. It encompasses public health measures for disease prevention as well as managing health crises like epidemics and natural disasters, both for communicable and non-communicable diseases [3]. Despite the significant progress achieved in previous years, the HSTP addresses multiple Saudi health challenges. These challenges can be categorized into four main areas: public health, workforce size, capabilities and participation, effective delivery of quality healthcare, and financial sustainability of the healthcare system [3]. One of the primary causes contributing to these challenges is that the current healthcare model prioritizes treatment over prevention, devoting significant resources to dealing with and treating diseases rather than concentrating on preventing them through preventive approaches [3]. For instance, each year, approximately 90,000 individuals suffer premature deaths from chronic diseases, with a notable increase in diabetes expected to double by 2030. Moreover, the alarming frequency of deaths and injuries due to traffic accidents results in substantial human, societal, and economic impacts. Therefore, it is crucial to prioritize preventive healthcare measures aimed at addressing a wide spectrum of health concerns, including heart disease, stroke, diabetes, respiratory illnesses, mental health disorders, congenital conditions, and traffic accidents [3].

This highlights the pivotal role of public health and preventive medicine physicians in achieving the strategic health objectives of the HSTP. They are instrumental not only in preventing diseases but also in policy development, community engagement, quality improvement, and health promotion efforts. Therefore, this study aimed to assess the levels of awareness and knowledge among the public health and preventive medicine physicians in Saudi Arabia towards the national healthcare transformation program.

## Materials and methods

Study design and study population

This is a cross-sectional study, which was conducted among public health and preventive medicine physicians working in Saudi Arabia, with eligibility extending to residents, specialists, and consultants.

Sample size 

The sample size is estimated at 270, calculated using Raosoft, Inc. Sample Size Calculator (Raosoft Inc., Seattle, WA), based on the population size of 903 public health and preventive medicine physicians as per the latest report of the Saudi Ministry of Health in 2022, with a 95% confidence interval, a 5% margin of error, and a response distribution estimated at 50%.

Sampling and data collection approach

Participants were recruited through convenience sampling, primarily via professional groups on WhatsApp (Meta Platforms, Inc., Menlo Park, CA), a platform widely used by the medical community in Saudi Arabia. Physicians completed the questionnaire voluntarily using Google Forms, which was distributed through WhatsApp. They could respond independently and anonymously. The survey, designed to assess awareness and knowledge of key health initiatives, took approximately three to five minutes to complete.

Data collection

A newly developed and validated self-administered questionnaire was used to collect data on physicians' awareness and knowledge of the national healthcare transformation program. Data collection started from April 28, 2024, to June 8, 2024. The initial draft was refined with input from subject matter experts and a biostatistician to ensure reliability and validity. The survey questionnaire included three sections: background characteristics, such as age, gender, region, affiliation, job rank, and experience; measurement of public health and preventive medicine physicians' awareness of the HSTP; and their confidence in their awareness, rated on a five-point Likert scale; measurement of public health and preventive medicine physicians' knowledge of the HSTP through six multiple-choice questions, with each correct answer receiving one point, leading to a total score out of six. This total knowledge score was used to determine the association between demographic data and knowledge levels.

The survey assessed the general awareness and knowledge of public health and preventive medicine physicians regarding the HSTP, its strategic objectives, the new Model of Care (MOC), value-based healthcare, Accountable Care Organizations (ACOs), and population health.

Ethical approval

The study was approved by the institutional review board (IRB) of King Fahad Medical City, Riyadh, Saudi Arabia (reference number 24-099C). Participants provided informed consent prior to their inclusion in the study, and their confidentiality was maintained.

Statistical analysis 

Data were analyzed using the IBM SPSS Statistics software for Windows, version 27 (IBM Corp., Armonk, NY). Descriptive statistical measures for qualitative categorical variables were frequency and percentage, and for quantitative data, they were mean ± standard deviation. Student's t-test and one-way ANOVA test were used to examine the association between knowledge score and background characteristics of the study participants. The multivariable linear regression was used to determine the predictors impacting the knowledge of public health and preventive medicine physicians towards the national healthcare transformation program. Statistical significance was set at a p-value of less than 0.05.

## Results

Participants' background characteristics

In this study, 307 public health and preventive medicine physicians in Saudi Arabia were included. Their mean age ± SD was 33.1 ± 5.1 years, and 69.4% of them were between 25 and 35 years. Among the participants, 54.1% were male, 40.1% lived in the Western region, and 25.7% lived in the Central region. Additionally, 76.2% of the physicians worked in the Ministry of Health, and more than half (54.7%) were public health and preventive medicine residents. Most participants (60.9%) had 0 to five years of work experience, while only 1.6% had over 20 years of experience (Table 1).

**Table 1:** Background characteristics of the study participants Age mean ± SD = 33.1 ± 5.1

Variables	Category	Frequency	Percentage (%)
Age (in years)			
	25-34	213	69.4
	35-44	81	26.4
	45-55	13	4.2
Gender			
	Male	166	54.1
	Female	141	45.9
Geographical region			
	Central	79	25.7
	Eastern	33	10.7
	Western	123	40.1
	Northern	19	6.2
	Southern	53	17.3
Affiliation			
	Ministry of Health	234	76.2
	Ministry of National Guard	12	3.9
	Ministry of Defense	24	7.8
	Ministry of Education (Universities)	28	9.1
	Other	9	2.9%
Job rank			
	Resident	168	54.7
	Specialist	102	33.2
	Consultant	37	12.1
Experience			
	0-5 years	187	60.9
	6-10 years	77	25.1
	11-15 years	30	9.8
	16-20 years	8	2.6
	>20 years	5	1.6

Public health and preventive medicine physicians’ awareness of the HSTP

When assessing participants' awareness of the HSTP in Saudi Arabia, it was found that 96.4% of the participants had heard about the program (Table 2). Among the participants, 83.7% were informed about the program's strategic objective, which is to promote the prevention of health risks. In addition, 72% were familiar with the "Keep Well" system in the new MOC, 86% were aware of population health management, and 80.5% had heard about value-based healthcare (Table 2).

**Table 2:** Awareness of the public health and preventive medicine physicians towards the National Health Sector Transformation Program (HSTP)

Awareness	Frequency	Percentage (%)	95% confidence interval	
			Lower bound	Upper bound
Health Sector Transformation Program				
No	9	2.9	1.3	5.5
Yes	296	96.4	93.7	98.2
Not sure	2	0.7	0.1	2.3
Is the Health Sector Transformation Program’s strategic objective "promoting prevention of health risks"?				
No	24	7.8	5.1	11.4
Yes	257	83.7	79.1	87.7
Not sure	26	8.5	5.6	12.2
"Keep Well" system in the new Model of Care				
No	54	17.6	13.5	22.3
Yes	221	72.0	66.6	76.9
Not sure	32	10.4	7.2	14.4
Population health management				
No	27	8.8	5.9	12.5
Yes	264	86.0	81.6	89.7
Not sure	16	5.2	3.0	8.3
Value-based healthcare				
No	36	11.7	8.3	15.9
Yes	247	80.5	75.6	84.7
Not sure	24	7.8	5.1	11.4

For further assessment of the participants' awareness of the HSTP, they were asked to rate their confidence in how well they knew about the program. In each item, more than half of the participants expressed low to neutral confidence in their awareness (ranging from strongly disagree to neutral on a five-point Likert scale): knowledge of the HSTP(60.6%); understanding of the MOC (54%); familiarity with the Keep Well system (63.8%); awareness of population health management (52.5%); and knowledge about value-based healthcare (53.4%) (Table 3).

**Table 3:** Public health and preventive medicine physicians’ confidence in their awareness of the National Health Sector Transformation Program (HSTP) Note: The interpretation of the Likert scale mean score was as follows: 1.0-2.4, disagree; 2.5-3.4, neutral; and 3.5-5.0, agree.

Rate how much this statement applies to you	Strongly disagree (n, %)	Disagree (n, %)	Undecided (Neutral) (n, %)	Agree (n, %)	Strongly agree (n, %)	Mean ± SD
I feel confident that I know the Health Sector Transformation Program very well	19 (6.2%)	48 (15.6%)	119 (38.8%)	82 (26.7%)	39 (12.7%)	3.24 ± 1.1
I feel confident that I know the new Model of Care very well	24 (7.8%)	43 (14%)	99 (32.2%)	94 (30.6%)	47 (15.3%)	3.32 ± 1.1
I feel confident that I know “Keep Well" system very well	47 (15.3%)	60 (19.5%)	89 (29%)	71 (23.1%)	40 (13%)	2.99 ± 1.2
I feel confident that I know population health management very well	31 (10.1%)	38 (12.4%)	92 (30%)	89 (29%)	57 (18.6%)	3.34 ± 1.2
I feel confident that I know value-based healthcare very well	32 (10.4%)	46 (15%)	89 (28%)	81 (26.4%)	62 (20.2%)	3.31 ± 1.2

Public health and preventive medicine physicians’ knowledge of the HSTP

Most participants correctly identified the knowledge questions about the HSTP; 76.9% of the participants correctly defined value-based healthcare as a healthcare delivery model in which providers, including hospitals and physicians, are paid based on patient health outcomes, and 70.4% knew that privatizing the health sector is not one of the strategic objectives of the HSTP. Additionally, 76.9% correctly identified that the new model of care comprises the Six Systems of Care (SOC): Keep Well, Safe Birth, Planned Care, Urgent Care, Chronic Care, and Last Phase. Moreover, 72.6% of participants correctly defined population health as the distribution of health outcomes of a defined group of people, the determinants that influence the distribution, and the policies and interventions that affect the determinants. About half of the participants (50.2%) knew that accountable care organizations would be the developed form of health clusters. Furthermore, 47.2% correctly chose that premarital screening was not a part of the Keep Well system (Table 4).

**Table 4:** Knowledge of the public health and preventive medicine physicians towards the National Health Sector Transformation Program (HSTP)

Knowledge assessment	Frequency	Percentage (%)
What does the following statement define? “A healthcare delivery model in which providers, including hospitals and physicians, are paid based on patient health outcomes”		
Right answer (Value-based healthcare)	236	76.9
Wrong answer	71	23.1
Health Sector Transformation Program includes all these strategic objectives, except?		
Right answer (Privatize the health sector)	216	70.4
Wrong answer	91	29.6
What do the following comprise? "Keep Well, Safe Birth, Planned Care, Urgent Care, Chronic Care, and Last Phase".		
Right answer (New Model of Care)	236	76.9
Wrong answer	71	23.1
Accountable Care Organizations will be the developed form of:		
Right answer (Health clusters)	154	50.2
Wrong answer	153	49.8
What does the following statement define? “The distribution of health outcomes of a defined group of people, the determinants that influence the distribution, and the policies and interventions that affect the determinants.”		
Right answer (Population health)	223	72.6
Wrong answer	84	27.4
All these initiatives are part of the Keep Well system, except?		
Right answer (Premarital screening)	145	47.2
Wrong answer	162	52.8

Association between knowledge score and background characteristics

Regarding the associations between knowledge scores and various background characteristics among public health and preventive medicine physicians, we found that physicians aged 45 to 55 years had notably higher knowledge scores than their younger counterparts. Additionally, residents of the Central region scored significantly higher than physicians from the Western region. Moreover, resident physicians had lower knowledge scores compared to specialist and consultant physicians. Another noteworthy finding was that physicians with 16 to 20 years of work experience exhibited significantly higher knowledge scores in comparison to those with 0 to five years of experience. Notably, we did not observe any significant differences in knowledge scores based on gender or affiliation (Table 5).

**Table 5:** Association between the knowledge scores and background characteristics of the study participants, i.e., the public health and preventive medicine physicians p-value based on one-way ANOVA *significant; **highly significant

Background characteristics		Knowledge score (0-6) = mean±SD	p-value
Age group	25 - <35 years	3.8±1.5	<0.001**
	35 - <45 years	4.1±1.4	
	45 - <55 years	5.2±0.8	
Gender	Female	3.8±1.4	0.106
	Male	4.1±1.5	
Geographical region	Central	4.3±1.4	0.031*
	Eastern	3.8±1.7	
	Western	3.7±1.5	
	Northern	4.4±1.2	
	Southern	3.9±1.4	
Affiliation	Ministry of Health	3.8±1.5	0.18
	Ministry of National Guard	4.5±1.6	
	Ministry of Defense	3.9±1.4	
	Ministry of Education (Universities)	4.5±1.1	
	Other	4.9±1.3	
Job rank	Resident	3.5±1.5	<0.001**
	Specialist	4.3±1.4	
	Consultant	4.8±1.1	
Experience	0-5 years	3.7±1.5	0.002*
	6-10 years	4.2±1.5	
	11-15 years	4.2±1.3	
	16-20 years	5.2±1.1	
	>20 years	4.8±1.1	

Factors affecting the knowledge of public health and preventive medicine physicians towards the HSTP

The multivariable linear regression model revealed that 16.4% of the variation in knowledge scores towards HSTP can be attributed to factors such as age, gender, affiliation, geographical region, job rank, and work experience. The analysis identified job rank as a significant predictor, with specialists and consultants having significantly higher knowledge scores compared to residents. Specialists had a knowledge score that was 0.669 points higher than residents (p <0.001), while consultants had a knowledge score that was 0.985 points higher than residents (p = 0.003).

It has been identified that work experience of 16-20 years contributed significantly to higher knowledge scores, 1.420 points more, compared to those with 0 to five years of experience. Other work experience categories (six to 10 years, 11 to 15 years, and >20 years) do not show statistically significant differences in knowledge scores compared to the 0 to five-year reference group.

Regarding geographical regions, only the Western region showed significantly lower knowledge scores compared to the Central region (B = -0.550, p = 0.008). Other regions (Eastern, Northern, and Southern) did not show statistically significant differences in knowledge scores compared to the Central region (Table 6).

**Table 6:** Identification of factors affecting the knowledge of public health physicians towards the National Health Sector Transformation Program (HSTP) *significant; **highly significant

Knowledge score	Unstandardized coefficients		Standardized coefficients	t	p-value	95.0% Confidence interval for B	
	B	Std. Error	Beta			Lower Bound	Upper Bound
Age (in years)	-.034	.030	-.116	-1.117	.265	-.093	.026
Gender (Reference: females)							
Males	.200	.173	.068	1.159	.248	-.140	.540
Affiliation (Reference: Ministry of Health)							
Ministry of National Guard	.715	.416	.095	1.720	.087	-.103	1.533
Ministry of Defense	-.144	.313	-.026	-.461	.645	-.760	.472
Ministry of Education	.124	.303	.024	.408	.684	-.473	.720
Other	.417	.487	.048	.857	.392	-.542	1.377
Work experience (Reference: 0-5 years)							
6-10 years	.402	.221	.119	1.822	.070	-.032	.837
11-15 years	.509	.359	.103	1.417	.157	-.198	1.216
16-20 years	1.420	.669	.154	2.121	0.035*	.102	2.737
>20 years	.910	.870	.079	1.046	.296	-.802	2.622
Geographical region (Reference: Central region)							
Eastern	-.288	.297	-.061	-.968	.334	-.873	.297
Western	-.550	.207	-.184	-2.652	0.008*	-.958	-.142
Northern	.374	.368	.061	1.016	.310	-.350	1.098
Southern	-.304	.252	-.078	-1.204	.229	-.800	.192
Job rank (Reference: Resident)							
Specialist	.669	.190	.215	3.531	<0.001**	.296	1.042
Consultant	.985	.326	.219	3.021	.003*	.343	1.627
(Constant)	4.639	.903		5.136	<0.001**	2.862	6.417

## Discussion

The HSTP seeks to reform the Saudi health sector, improving its effectiveness and integration into a patient-centered ecosystem that emphasizes value. Additionally, the HSTP aims to promote transparency and financial stability through initiatives focused on public health, disease prevention, and the adoption of contemporary healthcare models. Despite the significant progress achieved in previous years, major challenges still exist, and the public health sector is one of these challenges [3].

The crucial role of informed and well-prepared public health and preventive medicine physicians becomes apparent here. They serve as the cornerstone for population health by assessing the health of communities, developing robust public health policies based on scientific evidence and effective leadership, and ensuring that essential safety-net health services are available to those in need [5]. 

Public health and preventive medicine physicians in this study demonstrated a high level of awareness about the HSTP, mirroring findings from studies among healthcare workers in Tabuk and the Riyadh First Health Cluster staff [6, 7]. Specifically, 70.4% of our participants correctly identified the HSTP's strategic objectives. However, many expressed uncertainty when rating their confidence in their knowledge of the program. The uncertainty in physicians' understanding of the HSTP can stem from several factors: insufficient training and education; physicians' busy schedules and heavy workloads may limit their ability to stay informed about the details of the reform program; reliance on inconsistent and unofficial information sources. For instance, Ghalibi et al. found that although 71.5% of healthcare workers correctly identified the program's objectives, only 9.6% used official channels for information, while 72.4% relied on social media [6]. Additionally, a lack of engagement in the program's development and cognitive and emotional resistance to new policies and practices can lead to selective absorption of information.

Addressing these issues through targeted interventions can significantly improve physicians' comprehensive understanding and engagement with the program. This can be achieved by integrating the program into the curricula of medical and health colleges, offering intensive interactive training courses across various medical sectors, and establishing official communication channels to provide easy access to accurate information about the program. Ensuring that healthcare workers, who are key stakeholders in implementing the HSTP, have comprehensive knowledge of the program. A well-coordinated and informed healthcare workforce will facilitate the transformation agenda and strengthen the capacity of essential stakeholders in the process of change [8].

The new MOC is a key strategic reform initiative of the HSTP program that emphasizes the prevention of diseases and improves health awareness within the community. It aims to ensure access to health services through optimal coverage, equitable geographical distribution, comprehensive e-health services, and digital solutions. Additionally, the MOC focuses on the continuous improvement of health services by prioritizing beneficiaries' experience and satisfaction, adhering to international standards and best practices [4].

The current study results demonstrated that 72% of participants were aware of the MOC, and more than two-thirds correctly identified the MOC's SOC: keeping well, planned procedures, women & children, urgent problems, chronic conditions, and the last phase of life [4]. These results are higher than those reported in the study by Ghalibi et al., where only 49% of healthcare workers correctly defined the MOC, with many displaying confusion or a lack of clarity about it [6]. Additionally, in the study by Alomari et al., only 21.4% of participants demonstrated a high understanding of the MOC main outcomes [7].

When asked about the "Keep Well" initiatives, in one of the MOC's SOC, less than half (47.2%) of our participants correctly identified the keeping well components. These components include the health coach program, community-based wellness programs, workplace wellness programs, school wellness programs, healthy food promotion, health edutainment programs, and promoting the Saudi CDC [4].

These findings highlight the need for more efforts to educate healthcare workers, especially public health and preventive medicine physicians, who should be the most knowledgeable about the MOC and its initiatives. Ensuring comprehensive understanding and implementation of all MOC interventions is crucial for streamlining the Saudi healthcare system in alignment with the Kingdom’s Vision 2030 [9].

Accountable care organizations are designed to leverage the natural clustering of healthcare services near hospitals, as indicated by analyses of regional service utilization patterns [10]. An ACO is described as a collaborative group of hospitals, physicians, and other providers collectively responsible for enhancing the health and well-being of a specific population under their accountability [11]. This objective is advanced by placing a greater focus on preventive care delivered through primary care services and by coordinating care across different levels of healthcare [12].

This study revealed significant confusion and uncertainty regarding the definition of ACOs. Only half of the participants correctly recognized ACOs as evolved forms of health clusters. In a study conducted by Alharbi et al. among physicians working in hospitals and primary healthcare centers in Saudi Arabia, findings showed that just 15.3% of participants had excellent knowledge of ACOs. Additionally, 75.4% had knowledge scores ranging from good to fair, while 35% of physicians lacked clarity on how, when, and which specialties to engage in order to implement the ACO model effectively in Saudi Arabia [13]. 

It is crucial to enhance healthcare workers' awareness and knowledge of the ACO system, as the success of an ACO relies heavily on the leadership of proactive healthcare professionals who understand the broader context when taking initiative and making decisions [14]. Improving this awareness will lead to better healthcare service quality, reduced costs, positive perceptions, and minimized resistance toward achieving future visions for ACO settings [15].

Value-based healthcare is a key goal in Saudi Arabia's healthcare transformation agenda. It is a model where healthcare providers, such as hospitals and physicians, receive compensation based on the health outcomes of their patients. In this approach, providers are incentivized to assist patients in enhancing their health, minimizing the impact and occurrence of chronic diseases, and promoting healthier lifestyles through evidence-based practices [16]. 

In the present study, 80.5% of public health and preventive medicine physicians demonstrated awareness of value-based healthcare, with 76.9% providing accurate definitions. In contrast, a study involving 1,000 physicians in Brazil found that only 27% considered themselves highly aware of value-based healthcare [17]. The Oregon Academy of Family Physicians conducted a survey aimed at assessing family physicians' current knowledge and viewpoints on value-based care. The results indicated that 52% of respondents could precisely define value-based healthcare. Additionally, 31.3% of physicians acknowledged that value-based care contributes to enhanced patient care, while almost 27% identified improved compensation for primary care as a notable advantage of value-based care [18].

While the implementation of the value-based healthcare model faces several obstacles such as staffing shortages, inadequate healthcare software, and other resource limitations [19], the Council of Health Insurance in Saudi Arabia has made substantial strides. These include standardizing data and introducing a Minimum Data Set (MDS), launching a modern health information exchange platform (NPHIES), and implementing patient classification systems (AR-DRG and SBS). Furthermore, there has been an elevation in requirements and standards for health information management, encompassing clinical coding, training, accreditation, and billing [20].

Population health is the distribution of health outcomes within a specific group of individuals, the factors influencing this distribution, and the policies and interventions that impact those factors [21]. In this study, 72.6% of participants accurately defined population health.

This finding is encouraging, as population health, a longstanding concept, has gained substantial acceptance in the current era of healthcare reform. Understanding population health allows physicians to assess community health needs accurately, develop evidence-based policies, and implement effective prevention strategies. This knowledge helps in identifying and addressing health disparities, promoting equitable healthcare access, and optimizing resource allocation. By leveraging population health insights, public health, and preventive medicine physicians can ensure that the HSTP’s initiatives are tailored to the specific health challenges of different communities, thereby enhancing the overall impact and sustainability of the program [22].

Regarding the relationships between knowledge scores and various background characteristics among public health and preventive medicine physicians, we found that older physicians, consultant physicians, and those with more years of work experience exhibited higher knowledge scores compared to their peers. Furthermore, regression analysis revealed that job rank was the significant factor influencing public health and preventive medicine physicians' knowledge of the HSTP, with consultants demonstrating greater knowledge than specialists and residents. This aligns with findings from Alharbi et al., where consultants with over 10 years of clinical experience had the highest levels of knowledge about ACOs [13]. Similarly, Makdisse et al. reported that higher awareness of value-based healthcare was associated with older physicians aged 40 and above, as well as those in management positions [17]. This suggests that the HSTP and its concepts are still more intensively discussed at higher organizational levels and have not yet been fully disseminated to those less involved in organizational decision-making. Additionally, healthcare workers in Saudi Arabia require continuous development of skills and competence, starting from training through graduation and extending into workplace practices [23]. 

To our knowledge, this is the first study in Saudi Arabia concerned with the awareness and knowledge of public health and preventive medicine physicians toward the HSTP. Despite this, a few points of limitation must be considered. The study relies on self-reported data, which can be subject to bias. Participants might overestimate or underestimate their awareness and knowledge levels due to social desirability bias. Being a cross-sectional study, it captures information at a single point in time using a non-probability sampling technique, which might affect the generalizability of the results. This design does not account for changes in knowledge and awareness over time or the impact of recent initiatives and programs on healthcare workers' understanding of the HSTP. Addressing these limitations in future research could provide a more comprehensive understanding of the factors influencing public health and preventive medicine physicians' knowledge and awareness of the HSTP and contribute to more effective strategies for its implementation.

Recommendations

To enhance the implementation of the HSTP in Saudi Arabia, several key recommendations are proposed. These include integrating the HSTP concepts into medical and health college curricula and offering regular intensive training for healthcare workers. Improved information dissemination strategies involve establishing official communication channels for accurate outreach. Targeted professional development initiatives should focus on continuous education and mentorship programs for comprehensive understanding among healthcare professionals. Strengthening organizational support entails engaging all levels of healthcare organizations and fostering interdepartmental collaboration. Monitoring and evaluation efforts should regularly assess knowledge gaps and effectiveness, using feedback to tailor training and dissemination strategies. Additionally, promoting research and innovation will support ongoing improvements aligned with HSTP objectives and Vision 2030 goals.

## Conclusions

The HSTP in Saudi Arabia aims to revitalize the healthcare system by enhancing effectiveness, integrating patient-centered care, and emphasizing value. Despite progress, challenges remain, especially in the public health sector. This study shows that while public health and preventive medicine physicians are generally aware of the HSTP's objectives, many have inadequate confidence in their knowledge, highlighting the need for better information dissemination. Awareness of the MOC is high, but gaps persist in understanding specific components like the "Keep Well" initiative. Although value-based healthcare is well recognized, more effort is needed to fully understand and overcome the challenges of its implementation. Understanding population health is crucial for effective policymaking and the success of Vision 2030. The study also found that job rank and experience influence knowledge levels, suggesting the need for broader dissemination of the HSTP’s concepts.
